# High-Capacity Adenoviral Vectors Permit Robust and Versatile Testing of *DMD* Gene Repair Tools and Strategies in Human Cells

**DOI:** 10.3390/cells9040869

**Published:** 2020-04-02

**Authors:** Marcella Brescia, Josephine M. Janssen, Jin Liu, Manuel A. F. V. Gonçalves

**Affiliations:** Department of Cell and Chemical Biology, Leiden University Medical Center, Einthovenweg 20, 2333 ZC Leiden, The Netherlands

**Keywords:** gene editing, gene repair, CRISPR-Cas9, multiplexing, high-specificity nucleases, high-capacity adenoviral vectors, retargeting, Duchenne muscular dystrophy

## Abstract

Duchenne muscular dystrophy (DMD) is a fatal X-linked muscle wasting disorder arising from mutations in the ~2.4 Mb dystrophin-encoding *DMD* gene. RNA-guided CRISPR-Cas9 nucleases (RGNs) are opening new DMD therapeutic routes whose bottlenecks include delivering sizable RGN complexes for assessing their effects on human genomes and testing ex vivo and in vivo *DMD*-correcting strategies. Here, high-capacity adenoviral vectors (HC-AdVs) encoding single or dual high-specificity RGNs with optimized components were investigated for permanently repairing defective *DMD* alleles either through exon 51-targeted indel formation or major mutational hotspot excision (>500 kb), respectively. Firstly, we establish that, at high doses, third-generation HC-AdVs lacking all viral genes are significantly less cytotoxic than second-generation adenoviral vectors deleted in *E1* and *E2A*. Secondly, we demonstrate that genetically retargeted HC-AdVs can correct up to 42% ± 13% of defective *DMD* alleles in muscle cell populations through targeted removal of the major mutational hotspot, in which over 60% of frame-shifting large deletions locate. Both *DMD* gene repair strategies tested readily led to the detection of Becker-like dystrophins in unselected muscle cell populations, leading to the restoration of β-dystroglycan at the plasmalemma of differentiated muscle cells. Hence, HC-AdVs permit the effective assessment of *DMD* gene-editing tools and strategies in dystrophin-defective human cells while broadening the gamut of *DMD*-correcting agents.

## 1. Introduction

Duchenne muscular dystrophy (DMD; MIM #310200) is amongst the most common monogenetic disorders, affecting ~1 in 4700 boys [1]. This lethal X-linked muscle wasting disease is caused by loss-of-function mutations in the very large (~2.4 Mb) dystrophin-encoding *DMD* gene [2,3]. The largest dystrophin isoform (427 kDa) is translated from an 11-kb coding sequence embedded in a 14 kb mRNA transcript. This protein anchors the cytoskeleton to the dystrophin-associated glycoprotein complex (DGC) located along the sarcolemma of striated muscle cells [4]. Components of the DGC, including dystroglycans, sarcoglycans, sarcospan, dystrobrevins, syntrophin and nNOS, are not properly assembled in the absence of dystrophin [5]. This leads to a cascade of adverse events involving sarcolemma instability, impaired cell signaling and contractile dysfunction. These processes result in muscle necrosis, inflammatory cell infiltration and, eventually, replacement of functional muscle by fibrotic and adipose tissues. As a consequence, patients are usually wheelchair-bound around 12 years of age and commonly die in their thirties due to respiratory or cardiac failure [5].

DMD-causing mutations include point mutations, small rearrangements, duplications and, most frequently, frame-shifting large deletions [6]. Amongst the large deletions and large duplications, 66% and 15%, respectively, locate between exons 45 and 55, which constitutes a so-called major mutational hotspot region [6]. *DMD* deletions that do not disrupt the reading frame give rise instead to internally truncated, yet partially functional, dystrophins, which underlie Becker muscular dystrophy [7] (BMD; MIM #300376). As BMD patients often present mild muscle weakness and longer life expectancies [7], ongoing major efforts are directed towards endowing DMD patients with a BMD-like phenotype through RNA-level exon skipping, microdystrophin gene replacement and, more recently, gene editing [5,8].

Gene editing based on RNA-guided CRISPR-Cas9 nucleases (RGNs) is opening up the possibility for correcting disease-causing mutations such as those in the *DMD* gene [5,8,9]. These nucleases are ribonucleoprotein complexes consisting of a Cas9 endonuclease and a single guide RNA (gRNA). The Cas9 protein cleaves target sequences composed of a protospacer adjacent motif (PAM) located next to a 20 bp sequence complementary to the 5′ end of the gRNA [10,11,12]. The prototypic and commonly used *Streptococcus pyogenes* Cas9 (158-kDa) is encoded by a sizable open reading frame (4.1 kb) and has NGG as its PAM [10,11,13]. Targeted double-stranded DNA breaks (DSBs) induced by RGNs activate the non-homologous end-joining (NHEJ) pathway. The prevalence and operationality of NHEJ in dividing and post-mitotic mammalian cells renders it appealing for gene-editing purposes [11,13].

Initial NHEJ-based *DMD* gene editing experiments involved generating small insertions and deletions (indels) for resetting the reading frame directly or upon splice motif disruptions leading to exon-skipping or targeted DNA deletions [14,15,16,17]. These initial studies provided key proof-of-principles for NHEJ-mediated *DMD* repair in muscle progenitor cells and pluripotent stem cells. However, due to the relatively low efficiencies in gene-editing tool delivery, these experiments invariably relied on selection procedures or clonal isolations prior to the detection of Becker-like dystrophins. Hence, regardless of the specific *DMD* gene-editing approach, crucial developments are in demand especially at the levels of delivering and optimizing the necessary gene-editing tools. In this context, viral vector-mediated transfer of RGNs into dystrophic animal models and *DMD*-defective human myogenic cells is expanding the range of candidate in vivo and ex vivo DMD genetic therapies [18]. Adenoviral vectors (AdVs) [19] and adeno-associated viral vectors (AAVs) [20] are proving to be efficient vehicles for introducing gene-editing tools into human myogenic cells [21,22] and dystrophic animal models [23,24,25,26]. However, AAVs, with their limited packaging capacity (~4.7 kb) cannot transfer expression units encoding *S. pyogenes* multiplexing RGN pairs, i.e., dual RGNs. Moreover, consistent with earlier data showing that AAV DNA is prone to homology-independent insertion (“capture”) at random and targeted chromosomal DSBs in vitro [27,28,29], more recent data indicate that AAV transduction of programmable nucleases leads to high-frequency AAV DNA capture at target sequences in vivo as well [30,31], including in RGN-treated DMD mouse models [32,33]. First-generation *E1*-deleted AdVs, on the other hand, induce robust innate and adaptive immune responses in vivo and, at high doses, cytotoxicity in vitro due to “leaky” viral gene expression [19]. Moreover, human muscle progenitor cells as well as non-muscle cells with myogenic capacity, e.g., mesenchymal stromal cells, lack the Coxsackie B virus and Adenovirus receptor (CAR) engaged by prototypic AdVs based on species C human adenoviruses (i.e., types 2 and 5) [34,35,36,37]. These cells present instead on their surface ubiquitously expressed CD46, which is a receptor for species B human adenoviruses (e.g., types 35 and 50) [35,36,37,38]. Thus, endowing AdV particles with CD46-binding fiber motifs derived from species B adenoviruses is proving to be an effective genetic retargeting strategy for bypassing the absence of CAR on many cell types with high scientific and/or therapeutic relevance [35,36,37,38,39,40].

High-capacity AdVs (HC-AdVs), also known as “gutless”, helper-dependent or third-generation AdVs lack all viral genes, rendering them significantly less cytotoxic and immunogenic than first-generation AdVs [19,41,42,43]. Moreover, the possibility for packaging up to ~36 kb of foreign DNA in HC-AdV capsids permits transferring large expression units, including those encoding RGN multiplexes, into difficult-to-transfect cells [44,45].

Second-generation AdVs lacking *E1* and *E2A* are substantially more crippled than their first-generation counterparts [19]. *E2A* encodes a DNA-binding protein (DBP) which, in addition to assisting in trans-activating the viral gene expression program, is fundamental for viral DNA replication by cooperatively binding to single-stranded replicative intermediates [19]. In this study, we demonstrate that HC-AdVs are significantly less cytotoxic than AdVs deleted in *E1* and *E2A*. Moreover, we establish CD46-targeting HC-AdVs as efficient and versatile vehicles for testing *DMD* gene repair protocols by introducing into CAR-negative cells high-specificity RGNs encoding optimized Cas9 and gRNA components, either in single or multiplexing formats.

## 2. Materials and Methods

### 2.1. Cells

The origins of and culture conditions for the human wild-type myoblasts as well as for the *DMD*-defective myoblasts harboring *DMD* intragenic deletions Δ48–50 and Δ45–52, herein referred to as myoblasts DMD.Δ48–50 and DMD.Δ45–52, respectively, have been detailed elsewhere [46]. In brief, these cells were kept in Skeletal Muscle Cell Growth Medium (Ready-to-use, PromoCell; Cat. Nr.: C-23060) supplemented with 20% FBS, Glutamax (ThermoFisher Scientific; Cat. Nr.: 35050-061) and 1× Penicillin/Streptomycin (ThermoFisher Scientific; Cat. Nr.: 15140-122) at 37 °C in a humidified-air 5% CO_2_ atmosphere. The human cervix carcinoma HeLa cells (American Type Culture Collection) were cultured in high-glucose Dulbecco’s modified Eagle’s medium (DMEM; ThermoFisher Scientific; Cat. Nr.: 41966-029) supplemented with 5% fetal bovine serum (FBS; GE Healthcare Hyclone; Cat. Nr.: CH30160.03). The HC-AdV packaging cell line PEC3.30, derived from adenovirus type-5 *E1*-complementing PER.C6 cells [47], was cultured in high-glucose DMEM containing 10% FBS, 10 mM MgCl_2_ and 0.4 µg ml^−1^ puromycin. During vector production, the latter reagent was not included in the medium. The HeLa and PEC3.30 cells were kept at 37 °C and 39 °C, respectively, in a humidified-air 10% CO_2_ atmosphere. The PEC3.30 cell line was obtained after puromycin selection (0.5 µg ml^−1^) of a single cell-derived PER.C6 clone stably transfected with construct AH02_pCE.E2A-ts125.RSV.Cre.IRES.PuroR (Supplementary Information). PEC3.30 cells express the bacteriophage P1 site-specific Cre recombinase and a mutant form of the human adenovirus type-5 DNA-binding protein (DBP) encoded by the *E2A-ts125* open reading frame [48,49]. The product encoded by *E2A-ts125* presents a thermosensitive phenotype resulting from a serine-to-proline substitution at amino acid position 413. In particular, this DBP mutant acquires a misfolded, non-functional conformation at 39 °C, which transits to a functional, properly folded conformation at 34 °C. To overcome the growth disadvantage of cells constitutively expressing the wild-type DBP, research studies have resorted to this temperature switch for establishing stable *E1*- and *E2A*-complementing AdV packaging cells [50]. Hence, PEC3.30 cells, similarly to PER.E2A cells [50], are transferred from 39 °C to 34 °C during the production of HC-AdVs and second-generation AdVs, respectively, as the temperature of 34 °C is fully permissive for vector DNA replication. In the case of HC-AdV production, the gene products provided by the helper AdV, together with the extra amounts of functional DBP present at 34 °C in the producer cells, support the amplification and packaging of HC-AdV genomes.

### 2.2. Production and Characterization of Adenoviral Vectors 

The production and characterization of the second-generation AdV.Δ2.EGFP vector, previously named AdV.Δ2.donor^S1^, have been detailed before [51]. The HC-AdV molecular clones used for generating the fiber-modified vectors HC-AdV.eCas9, HC-AdV.eCas9^gEX51^ and HC-AdV.eCas9^gIN43.gIN54^ were, respectively, AW71_pHC-AdV.eCas9, AW72_pHC-AdV.eCas9.gEX51 and AW70_pHC-AdV.eCas9.gIN43.gIN54. The expression units encoding *DMD*-targeting gRNAs gEX51, gIN43 and gIN54 contain optimized scaffolds [52]. Prior to their insertion into the respective HC-AdV molecular clones, these expression units were assembled by ligating pairs of annealed oligonucleotides (Supplementary Table S1) into the BveI-digested plasmid AY56_pUC.U6.opt-sgRNA.BveI-stuffer [53]. The annotated maps and relevant nucleotide sequences of all HC-AdV genomes are available in the Supplementary Information. 

The assembly of fiber-modified vectors HC-AdV.eCas9, HC-AdV.eCas9^gEX51^ and HC-AdV.eCas9^gIN43.gIN54^ was initiated by transfecting the respective MssI-digested HC-AdV molecular clones into PEC3.30 cells seeded one day before in 6-well plates at a density of 1.6 × 10^6^ cells per well (Greiner Bio-One). Transfection reactions consisted of 6.25–6.5 µg of each of these MssI-digested HC-AdV plasmids mixed with 150 mM NaCl to a final volume of 200 µL to which 21 µL of a 1 mg mL^−1^ solution of 25-kDa linear polyethylenimine (PEI; Polysciences) was added. After an incubation period for 6 to 8 h at 39 °C, the fiber-modified *E1*-deleted helper AdV vector AdV.SRα.LacZ.1.50 [54] was added in fresh medium at a multiplicity of infection (MOI) of 40 infectious units (IU) per cell. At this stage, the PEC3.30 cells were transferred to 34 °C so that, as aforementioned, the thermosensitive DBP expressed in these cells acquires a proper conformation. The gene products provided by the helper AdV, together with the extra amounts of functional DBP expressed by the producer cells, support the amplification and encapsidation of HC-AdV genomes. The assembly of helper AdV particles is selected against by specific Cre/loxP-mediated excision of the floxed packaging elements present in the helper AdV genomes. At 5 days post-transduction, the producer cells were harvested, and assembled HC-AdV particles were released by three cycles of freezing (liquid N_2_) and thawing (37 °C water bath). Next, cellular debris were removed by 10-min centrifugations at 2000× *g*, and the supernatants were collected. The vector particles present in the clarified supernatants were subsequently amplified via four rounds of propagation in increasing numbers of packaging cells co-transduced with helper AdV.SRα.LacZ.1.50. The fourth, largest-scale propagation step involved twenty T175-cm^2^ culture flasks (Greiner Bio-One), each containing 24 × 10^6^ producer cells incubated with 2.6–3.0 mL of clarified supernatants from the previous propagation rounds together with helper AdV.SRα.LacZ.1.50 at an MOI of 62.5 IU per cell. At 3 days post-transduction, the producer cells were harvested and the HC-AdV particles were purified by sequential block and continuous cesium chloride buoyant density ultracentrifugation followed by ultrafiltration. Reagents and procedures used for purifying and de-salting large-scale AdV batches are specified in detail elsewhere [54,55], as are the methods used in determining vector titers as well as in characterizing vector genomes packaged in purified AdV capsids are specified [54,55]. The production and characterization of the reporter vector HC-AdV.EGFP involved essentially the same procedures applied for the production and characterization of the vector set HC-AdV.eCas9, HC-AdV.eCas9^gEX51^ and HC-AdV.eCas9^gIN43.gIN54^, except that the HC-AdV molecular clone AD03_pHC-AdV.EGFP (Supplementary Information) was used and the incubation of the transfection mixture lasted overnight instead of 6–8 h.

### 2.3. Transduction Experiments

To assess the transduction efficiencies of EGFP-encoding AdV particles in HeLa cells and human myoblasts, 5 × 10^4^ cells per well were seeded in 24-well plates (Greiner Bio-One). The next day, the cells were exposed in 500-μL volumes to different MOIs of each of the indicated viral vectors. Mock-transduced HeLa cells served as negative controls. For the quantification of cell viability and apoptosis after AdV transduction, 4 × 10^4^ HeLa cells per well were seeded in 24-well plates instead. *DMD* gene editing experiments were initiated by seeding DMD.Δ45–52 and DMD.Δ48–50 myoblasts at a density of 5 × 10^4^ cells per well of 24-well plates (Greiner Bio-One). The next day, DMD.Δ45–52 myoblasts were exposed in 500 μL volumes to different MOIs of each of the viral vectors as indicated in the figures. Negative controls were provided by mock-transduced DMD.Δ45–52 and DMD.Δ48–50 myoblasts, while positive controls for dystrophin expression were provided by differentiated myoblasts isolated from a healthy donor [46].

### 2.4. Cell Viability Assays

The extent of metabolic activity in cultures of human myoblasts and HeLa cells upon transduction with second-generation AdVs versus HC-AdVs was assessed by measuring, through colorimetry, the conversion of the tetrazolium salt (WST-8) to formazan after applying the reagents and following the instructions provided in the CCK-8 kit (Boster Biological Technology; Cat. Nr.: AR1160). Briefly, target cells were seeded in wells of 24-well plates (Greiner-BioOne) and were subsequently transduced with several MOIs of either second-generation AdVs or HC-AdVs as described above. At 2 days (HeLa) or 3 days (myoblasts) post-transduction, the cells were trypsinized and 1:40 (HeLa) or 1:10 (myoblasts) of each of the cell suspensions was transferred into wells of 96-well plates (Greiner-BioOne) and incubated in regular growth medium. Twenty-four hours after cell sub-culturing, 10 µL of WST-8 solution was added to each well, after which the plates were incubated for an additional 4–5 h at 37 °C. The absorbance of the solution in each well was measured by a standard microplate reader at 450 nm. The absorbance values measured directly correlate with the amount of viable metabolically active cells present in the initial samples.

The frequencies of apoptotic cells were determined by using the Annexin-V PE apoptosis staining detection kit (Abcam; Cat. Nr.: ab14155). The reagents and instructions provided by the manufacturer were applied. In brief, target cells were transduced with different MOIs of second-generation AdV or HC-AdV particles. HeLa cells were trypsinized and resuspended in fresh culture medium 2 days post-transduction, after which the cells were incubated for 24 h before proceeding with the Annexin-V PE staining. Three days post-transduction, myoblasts and HeLa cells were trypsinized, centrifuged and resuspended in 300 µL of Binding Buffer followed by addition of 5 µL of Annexin-V PE. Mock-transduced cells were subjected to the same procedure. After a 7 min incubation period in the dark, the frequencies of the Annexin V-positive cells were determined by PE-directed flow cytometry. The CCK-8 assay in myoblasts was performed on samples derived from three independent biological replicates.

### 2.5. Live-Cell Light Microscopy

Two days post-transduction, HeLa cells were trypsinized, resuspended in fresh culture medium and subsequently placed back in the incubator for 24 h before imaging. The DNA dye Hoechst 33342 (Thermo Scientific Cat. Nr: 62249) was diluted 1:10.000 in the culture medium, and the cells were then incubated at 37 °C in 10% CO_2_ for 10 min. Total and transgene-positive cells present in randomly selected microscopy fields were detected through fluorescence microscopy directed to the DNA dye and EGFP, respectively, by using an Olympus IX51 inverse fluorescence microscope equipped with an XC30 Peltier CCD camera and the CellF software (both from Olympus). Myoblasts were transduced as described above, and at 3 days post-transduction, the myoblasts were tripsinized and transferred to wells of 24-well plates. Bright-field images were acquired 2 days after re-seeding the cells by using an inverted Leica DMi8 microscope equipped with a DFC 450c camera, 40 × objective and the LAS 4.8.0 software (both from Leica).

### 2.6. Flow Cytometry

The frequencies of cells positive for Annexin-V or EGFP were determined by using a BD LSR II flow cytometer (BD Biosciences). Cells corresponding to experimental negative controls were used for establishing the threshold levels corresponding to background fluorescence. A minimum of 10,000 viable single cells were analyzed in each sample. Flow cytometry data analyses were carried out with the aid of FlowJo 10.6.1 software (TreeStar).

### 2.7. qPCR Assays

The frequencies of *DMD* intragenic deletions were assessed by qPCR assays as previously described in detail [22]. Briefly, 3 days post-transduction, mock-transduced and HC-AdV-transduced myoblasts were sub-cultured, and total cellular DNA was extracted at 7 days post-transduction using the DNeasy Blood & Tissue Kit (Qiagen; Cat. Nr.: 69506) according to the manufacturer’s instructions. Next, 5 ng samples of genomic DNA were diluted in 20 μL reaction mixtures containing 1 × iQTM SYBR Green Supermix (Bio-Rad; Cat. Nr.: 1708882) and 150 nM of each primer. Parallel qPCR amplifications targeting DNA sequences located in intron 43 of the *DMD* locus provided for internal controls. The RGN-induced *DMD* deletion and internal control target sequences were amplified in triplicate for all genomic DNA samples. The qPCR assay primers were designed with the aid of the open-source Primer3 program (http://frodo.wi.mit.edu/), and their sequences are specified in Supplementary Table S2. The qPCR amplifications were carried in a CFX Connect Real-Time System (Bio-Rad) using the thermo-cycling protocols indicated in Supplementary Table S3. After each qPCR run, the baseline thresholds and the estimated target DNA copy numbers were auto-calculated by using the Bio-Rad CFX Manager 3.1 software. The copy numbers of alleles harboring long-range *DMD* deletions were determined on the basis of standard curves using serial dilutions, ranging from 2.26 × 10 to 2.26 × 10^5^ copies, of reference plasmid AV24_jDEL.I43-I54 containing a copy of the dual RGN-induced *DMD* junction [22]. For the internal control samples, standard curves were made on the basis of serial dilutions, ranging from 7.56 × 10 through 7.56 × 10^5^ copies, of reference plasmid AL05_pDMD [22]. The specificity of the primers for the internal control and the dual RGN-induced major *DMD* hotspot deletion was confirmed by agarose gel electrophoresis analyses of the respective qPCR amplicons (Supplementary Figure S1). The identity of the qPCR amplicons was further confirmed by Sanger sequencing. Finally, in order to calculate targeted DNA deletion frequencies, the copy numbers of allelic *DMD* deletions were normalized, on a per-sample basis, to the copy numbers of the internal control. The qPCR assays were performed on genomic DNA samples derived from three independent biological replicates with each sample subjected to three qPCR technical replicates.

### 2.8. Immunofluorescence Microscopy Analyses

DMD.Δ45-52 and DMD.Δ48–50 myoblasts were transduced, and at 3 days post-transduction, the inocula were discarded. Next, the cells were transferred in regular culture medium into wells of 24-well plates previously pre-coated with a 0.1% (*w/v*) gelatin solution (Sigma-Aldrich; Cat. Nr.: G13393). The differentiation and fusion of myoblasts into myotubes were triggered, when wells reached full confluency, by changing the growth medium to a differentiation mitogen-poor medium consisting of phenol-red-free DMEM (ThermoFisher Scientific; Cat. Nr.: 11880-028) supplemented with 100 µg mL^-1^ human holo-transferrin (Sigma-Aldrich; Cat. Nr.: T0665) and 10 µg mL^−1^ human insulin solution (Sigma-Aldrich; Cat. Nr.: I9278) supplemented with 1× Penicillin/Streptomycin (Life Technologies). Cultures containing myotubes derived from wild-type myoblasts served as positive controls for dystrophin expression, whereas negative controls were provided by unedited DMD.Δ45–52 and DMD.Δ48–50 myotubes.

The resulting cultures of myotubes bearing wild-type, Δ45–52 or Δ48–50 genotypes or edited *DMD* genotypes were fixed with 4% paraformaldehyde (PFA) for 15 min, permeabilized in 0.5% (*v/v*) Triton X-100 in TBS (50 mM Tris-HCl pH 7.5, 100 mM NaCl) at room temperature for 10 min and blocked for 2–4 h in Antibody Diluting Solution (Abdil) consisting of TBS, 0.1% Triton X-100, 2% bovine serum albumin and 0.1% sodium azide. Primary antibodies directed against the C-terminus of dystrophin (abcam; Cat. Nr.: ab15277) or β-dystroglycan (Santa Cruz Biotechnologies; NCL-b-DG; Cat. Nr.: sc-33702) were diluted 1:100 in Abdil and then incubated with the specimens overnight at 4 °C. After three washes of 5 min each with 0.1% Triton X-100 TBS solution, the specimens were incubated with conjugated secondary antibodies AlexaFluor 488 goat anti-mouse IgG (H+L) (Life Technologies; cat. Nr.: A11001) and Alexa Fluor 568 goat anti-rabbit IgG (H+L) (Life Technologies; Cat. Nr.: A11036) diluted 1:500 in Abdil for 45 min to 1 h in the dark at room temperature. The coverslips were washed thrice for 5 min per wash with 0.1% Triton X-100 TBS solution and were subsequently mounted with ProLong™ Gold Antifade Mounting reagent containing 4′,6-Diamidine-2′-phenylindole dihydrochloride (DAPI; ThermoFisher Scientific; Cat. Nr.: P36931). The differentiation stage of human muscle progenitor cells was also assessed by immunofluorescence microscopy analyses using primary antibodies directed against two skeletal muscle markers, i.e., sarcomeric α-actinin (Sigma, Cat Nr.: A7811 clone EA-53) and skeletal fast myosin (Sigma, Cat Nr.: M4276 clone MY-32), both diluted 1:100 in blocking solution. After incubation with these primary antibodies, the specimens were exposed to a goat anti-mouse IgG (H+L) secondary antibody conjugated to AlexaFluor 488 (Life Technologies; cat. Nr.: A11001) diluted 1:500. Undifferentiated myoblasts and differentiated myotubes derived from a healthy donor were used as negative and positive controls, respectively, in the immunofluorescence microscopy assays directed at the two aforesaid skeletal muscle markers. The details of the staining protocol are described above.

The expression of Cas9 in DMD.Δ45–52 and DMD.Δ48–50 myoblasts transduced with HC-AdVs was assessed at 3 days post-transduction. The transduction conditions used are specified under the transduction experiments section. The immunofluorescence staining protocol applied for the detection of eCas9.4NLS was the same as that described above except that the 1:100 diluted primary antibody was directed against *S. pyogenes* Cas9 (Abcam, Cat Nr.: 191468 clone 7A9-3A3). The AlexaFluor 488-conjugated goat anti-mouse IgG (H+L) antibody (Life Technologies; cat. Nr.: A11001), diluted 1:500, was applied as secondary antibody.

Finally, the fluorescence microscopy analyses were carried out with a 40× objective upright Leica SP8 confocal microscope (Leica microsystems) equipped with Leica hybrid detectors, HyD (Leica microsystems). The images were analyzed using the ImageJ software (NIH, US National Institutes of Health). This assay was performed on samples derived from three independent biological replicates.

### 2.9. Western Blot Analysis

DMD.Δ45–52 and DMD.Δ48–50 myoblasts were transduced and were subjected to the cell culture and differentiation conditions performed as described previously in the Immunofluorescence microscopy analyses section. After 4 to 5 days in differentiation medium, the myotube-containing cultures were lysed on ice for 30 min by incubation in 50 μL of RIPA buffer (Thermo Scientific; Cat. Nr.: 89900) supplemented with a protease inhibitor cocktail (cOmplete Mini, Sigma-Aldrich; Cat. Nr.: 11836153001). Protein quantification of each sample was carried out by performing the Pierce BCA Protein Assay Kit (Thermo Scientific, Cat. Nr: 23225), following the manufacturer’s instructions. Next, cell lysates corresponding to 30 µg of total protein were diluted in 4× sample buffer and 20× reducing agent (both from Bio-Rad; Cat. Nr.: 161-0791 and 161-0792, respectively) and incubated at 95 °C for 5 min. Protein samples and 15 μl of HiMark Prestained Protein Standard (Thermo Scientific) were loaded in a 3–8% Criterion XT Tris-Acetate precast gel (Bio-Rad: Cat. Nr.: 3450130). The polyacrylamide gel was then placed in an ice-cold Criterion Cell and was run in XT Tricine running buffer (Bio-Rad; Cat. Nr.: 1610790) in two phases. The first phase took 30 min at 75 V (0.07 A), and the second proceeded for 1.5 h at 150 V (0.12 A). Subsequently, the resolved proteins were transferred with the aid of a Trans-Blot Turbo Midi PVDF pack (Bio-Rad; Cat. Nr.: 1704157) and a Trans-Blot Turbo system (Bio-Rad) according to the manufacturer’s recommendations for high-molecular-weight proteins (2.5 A, 25 V, 10 min). PVDF membranes were blocked for 2 h at room temperature in Blocker Blotto in TBS (ThermoFrisher Scientific; Cat. Nr.: 37530). Next, the membranes were incubated with a rabbit polyclonal antibody directed against dystrophin (abcam; Cat. Nr.: ab15277) diluted 1:500 in Blocker Blotto or against α/β tubulin (CST; Cat. Nr.: 2148) diluted 1:5000 in Blocker Blotto. After an overnight incubation period at 4 °C, the membranes were washed thrice in TBST for 10 min per wash and were then incubated for 2 h at room temperature with an anti-rabbit IgG secondary antibody conjugated to horseradish peroxidase (IgG-HRP; Santa Cruz) diluted 1:5000 in Blocker Blotto or IRDye goat anti-rabbit 800CW (Licor) diluted 1:5000 in Blocker Blotto for 45 min at room temperature. Proteins were detected by using horseradish peroxidase substrate Pierce ECL2 (Thermo Scientific; Cat. Nr.: 80196) following the manufacturer’s specifications and Super RX-N X-Ray film (Fujifilm) or Odyssey Western Blot Detection. This assay was performed on samples derived from three independent biological replicates.

### 2.10. Statistical Analyses

Statistical analyses were done by using the GraphPad Prism 8.0.1 software on datasets derived from a minimum of three independent biological replicates done on different days. Each qPCR data point corresponds to mean values from three technical replicates. The statistical significances were calculated with the tests indicated in the figure legends. *p-*values lower than 0.05 were considered to be statistically significant.

## 3. Results

### 3.1. At High Doses HC-AdVs Are Significantly Less Cytotoxic Than Second-Generation AdVs

Although second-generation AdVs are more crippled than their first-generation counterparts (Supplementary Figure S2), leaky viral gene expression can still be detected at high vector doses [50], which may result in cytostatic and/or cytotoxic effects in vector-transduced cells. Hence, in the present work, we sought to investigate the feasibility and utility of using tropism-modified HC-AdVs for NHEJ-mediated *DMD* repair through “all-in-one” delivery of single or dual RGN complexes. Moreover, we incorporated in these fully viral gene-deleted AdV particles expression units encoding improved high-specificity RGN components. In particular, the high-specificity eSpCas9(1.1) nuclease [56] endowed with four nuclear localization signals, i.e., eCas9.4NLS [53] and gRNAs with an optimized scaffold (opt-gRNAs) [52]. eCas9.4NLS displays enhanced nuclear enrichment resulting in higher targeted DNA cleaving activities when compared to its parental eSpCas9(1.1) protein [53]; opt-gRNAs are expressed to higher amounts and presumably confer higher RGN stability than their conventional counterparts [52]. Hence, the eCas9.4NLS:opt-gRNA complexes used herein outperform conventional RGNs based on regular Cas9 and gRNAs with non-optimized scaffolds at the levels of activity and specificity [53].

**Figure 1:**
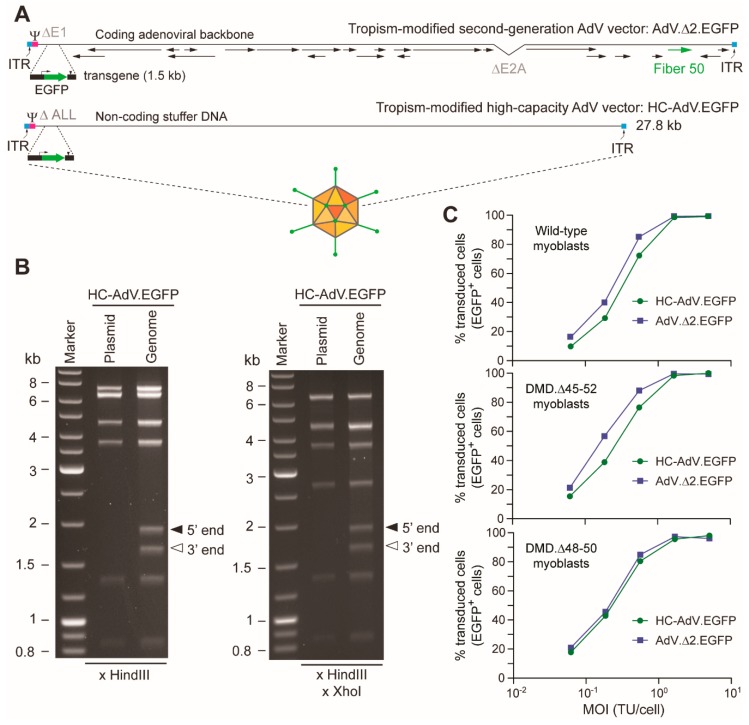
Characterization of high-capacity adenoviral vector (HC-AdV) DNA structure and transduction analysis on human myoblasts. (**A**) Diagrammatic representation of second-generation versus high-capacity AdV vector genomes. The second-generation AdV.Δ2.EGFP and the third-generation HC-AdV.EGFP have the reporter EGFP under the transcriptional control of the human *PGK1* gene promoter and the simian virus polyadenylation signal. In addition to the transgene, AdV.Δ2.EGFP harbors viral ORFs (some of which are indicated), whereas HC-AdV.EGFP contains non-coding “stuffer” DNA. ITR and Ψ: adenovirus type-5 cis-acting inverted terminal repeats and packaging signal necessary for vector DNA replication and encapsidation, respectively. (**B**) Determining HC-AdV genetic integrity. Restriction fragment length analyses were carried out by agarose gel electrophoreses of vector DNA extracted from purified vector particles and subsequently digested with HindIII alone or with HindIII and XhoI. Marker: GeneRuler DNA Ladder molecular weight mix (Fermentas). The parental plasmid pHC-AdV.EGFP treated with the same restriction enzymes served as an additional molecular weight reference. The two extra DNA fragments detected in the HC-AdV.EGFP lanes correspond to the 5′ (“left” end) and 3′ (“right” end) termini of the linear double-stranded vector genomes. (**C**) Gene transfer frequencies in human myoblast populations subjected to tropism-modified HC-AdV and second-generation AdV transductions. Transduction experiments in human muscle progenitors derived from different donors using HC-AdV.EGFP and AdV.Δ2.EGFP were assessed by EGFP-directed flow cytometry. A minimum of 10,000 viable single cells were analyzed per sample.

We started by generating pairs of second-generation AdVs and HC-AdVs displaying CD46-targeting fibers and encoding the same transgenes. These tropism-modified vector pairs encode either the EGFP reporter (i.e., AdV.Δ2.EGFP and HC-AdV.EGFP) or the eCas9.4NLS nuclease (i.e., AdV.Δ2.eCas9 and HC-AdV.eCas9). Structural analysis of recombinant HC-AdV.EGFP genomes (Figure 1A) isolated from a purified vector preparation established their genetic integrity with no evidence for species with rearrangements or truncations (Figure 1B).

**Figure 2:**
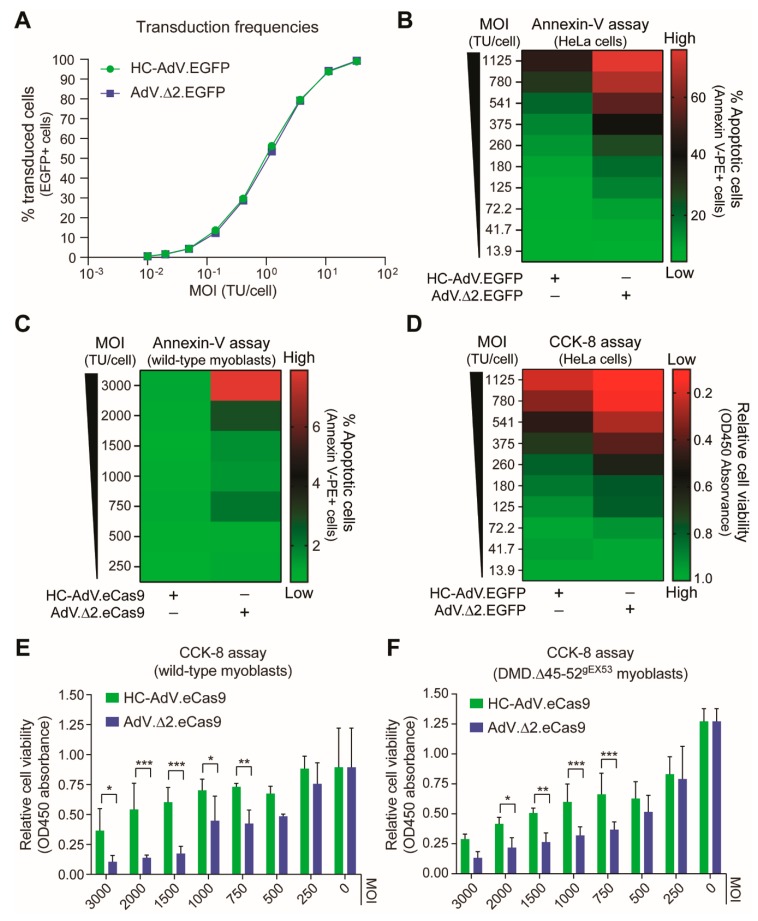
Comparing the cytotoxicity profiles of second-generation versus third-generation AdV particles in human cells. (**A**) Transduction efficiencies assessed by using EGFP-encoding AdV particles. Transduction of HeLa cells with different amounts of AdV.Δ2.EGFP or HC-AdV.EGFP, showing similar gene delivery activities for both vector types. (**B** and **C**) Quantification of apoptosis after AdV transductions. The apoptotic cell frequencies in HeLa cell and myoblast cultures exposed to different amounts of the indicated viral vectors were measured by annexin-V staining and flow cytometry. (**D**–**F**) Quantification of cell viability after AdV transductions. The cell viabilities in HeLa cell and myoblast cultures incubated with different amounts of the indicated viral vectors were determined by using the metabolic activity-sensing substrate CCK-8 and colorimetry. The various vector multiplicities of infection (MOI) are expressed as functional reporter transducing units per cell. Vector-transduced DMD.Δ45–52^gEX53^ myoblasts used in the experiments of panel **F** were subjected to DSBs as they express gRNA gEX53 that addresses *Streptococcus pyogenes* Cas9 nucleases to *DMD* exon 53. Significance among datasets (*n* = 3) was calculated by two-way ANOVA followed by Sidak’s test for multiple comparisons; *p < 0.05; **P < 0.01; ***p < 0.001; *p* ≥ 0.05 was considered non-significant.

**Figure 3:**
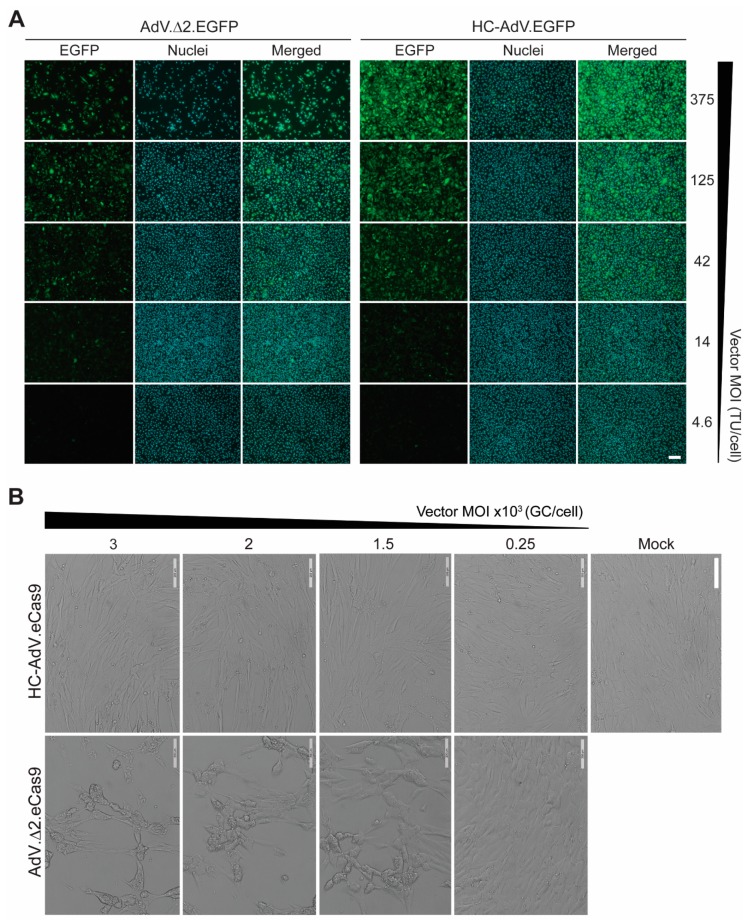
Comparing the impact of second-generation versus third-generation AdVs on human cells. (**A**) Direct fluorescence microscopy on HeLa cell cultures. HeLa cells were transduced with AdV.Δ2.EGFP or with HC-AdV.EGFP at the indicated multiplicities of infection (MOI) and were then subcultured at 2 days post-transduction. At 3 days post-transduction, total and transgene-positive cells present in randomly selected microscopy fields were detected through fluorescence microscopy directed to the DNA dye Hoechst 33342 and EGFP, respectively. TU/cell: transducing units per cell. (**B**) Bright-field microscopy on human myoblast cultures. Human DMD.Δ45–52 myoblasts were transduced with AdV.Δ2.EGFP or with HC-AdV.EGFP at the indicated multiplicities of infection (MOI) and were then subcultured at 3 days post-transduction. Bright-field microscopy images were acquired at 5 days post-transduction. GC/cell: genome copies per cell. Magnification bars correspond to 200 µm.

Dose–response transduction experiments on human myoblasts derived from a healthy donor and two DMD patients (i.e., DMD.Δ45-52 and DMD.Δ48-50 myoblasts) revealed that AdV.Δ 2.EGFP and HC-AdV.EGFP could achieve similar frequencies of EGFP-positive cells in a cell-donor-independent manner (up to 99%) (Figure 1C). Dose–response transduction experiments on human cervix carcinoma HeLa cells with AdV.Δ2.EGFP and HC-AdV.EGFP confirmed similar gene delivery activities for these two vector types (Figure 2A). Tellingly, however, dose–response transduction experiments on HeLa cells and human myoblasts using the panel of AdVs encoding EGFP or eCas9.4NLS revealed that second-generation AdVs are substantially more detrimental to cell viability than their HC-AdV counterparts. In particular, the frequencies of apoptotic cells were generally higher in cultures of HeLa cells and human myoblasts exposed to second-generation AdV particles (Figure 2B,C, respectively). In addition, metabolic activity measurements in HeLa cell cultures (Figure 2D) and in human myoblast cultures (Figure 2E,F) incubated either with second-generation AdVs or HC-AdVs, equally revealed that the former gene transfer system is generally more detrimental to cell viability than the latter. The results from these independent cell viability quantitative assays were corroborated by direct visual inspection of HeLa cells and human myoblasts exposed to each vector type in that an exacerbated dose-dependent decrease in cell numbers could readily be discerned after the transfer of cultures that had been incubated with second-generation AdVs (Figure 3).

### 3.2. HC-AdVs Encoding Single or Dual RGNs Induce Robust Dystrophin Synthesis in Unselected DMD Muscle Cell Populations

**Figure 4:**
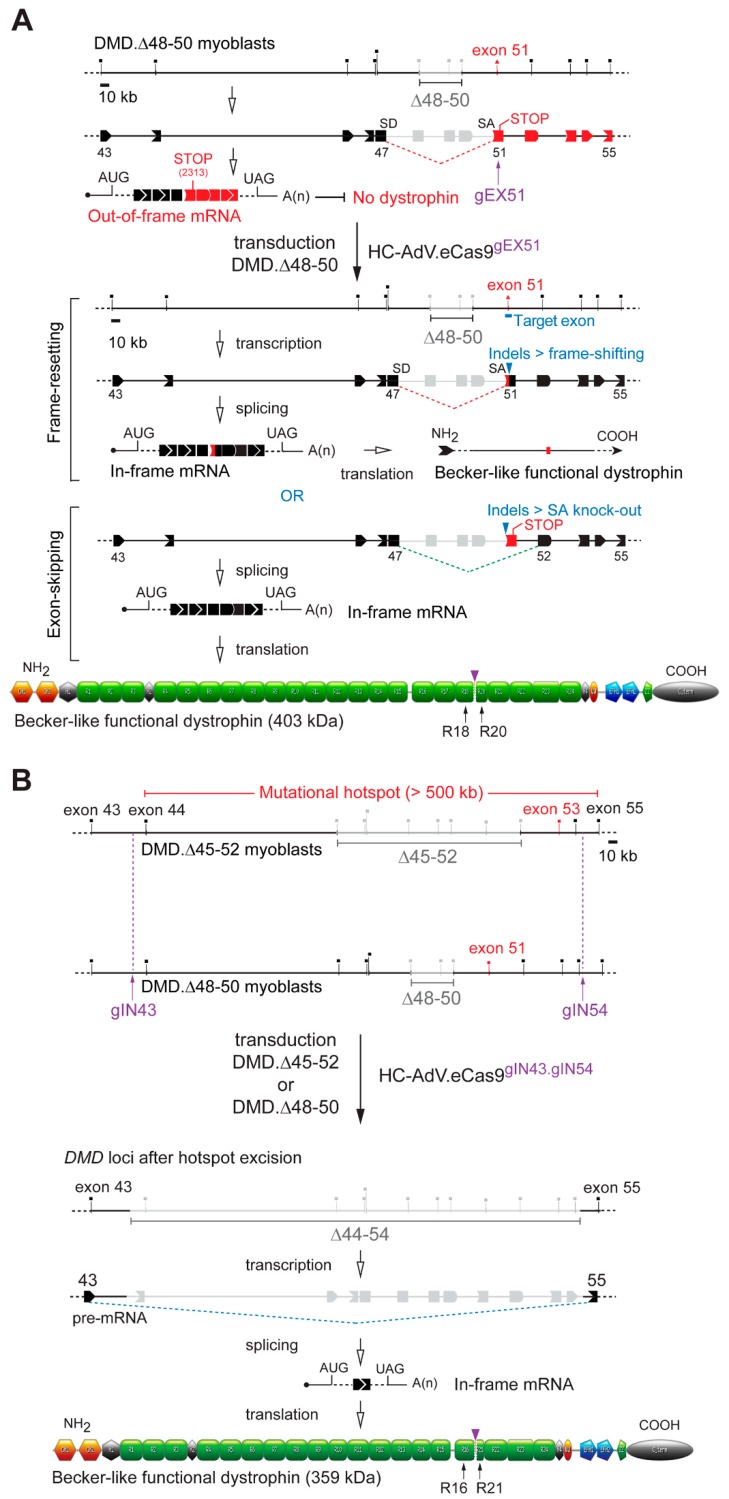
“All-in-one” HC-AdV transduction of RNA-guided CRISPR-Cas9 nucleases (RGNs) for *DMD* reading frame restoration. (**A**) *DMD* correction via NHEJ-induced reading frame resetting or exon-skipping. *DMD* deletions spanning exons 48 through 50 (i.e., *DMD* deletions Δ48–50) yield mRNA transcripts with frame shifts downstream of the exon 47–51 junction installing a premature stop codon. These transcripts result in low or no dystrophin in differentiated DMD.Δ48–50 muscle cells. HC-AdV-mediated delivery of RGNs cleaving genomic DNA between the exon 51 splice acceptor site (SA) and the downstream non-sense mutation (STOP) can install DSB-derived indels that yield Becker-like dystrophins either through reading frame resetting or SA knockout-induced exon-skipping. SD: splice donor site. (**B**) *DMD* correction via NHEJ-mediated multi-exon deletion. Removal of DMD-causing mutations (i.e., *DMD* deletions Δ45–52 and Δ48–50) in the major mutational hotspot after HC-AdV-mediated delivery of dual RGNs targeting introns 43 and 54. The excision of exons 44 through 54 results in the splicing of exon 43 to exon 55 and the generation of an in-frame mRNA species coding for a 359-kDa Becker-like dystrophin. Grey oval: C-terminal domain; green pentagon: ZZ zinc-finger domain; blue pentagons: EFH1 and EFH2 hand-regions harboring cysteine-rich motifs that, among other proteins, interact with β-dystroglycan; orange oval: WW domain, green boxes, spectrin-like repeats; black hexagons: hinges; orange hexagons: CH1 and CH2 actin-binding domain; vertical arrowheads: junctions between amino acids in the indicated spectrin-like repeats (R) after *DMD* gene editing. The diagrams showing the structural organization of the Becker-like dystrophins assembled after single and dual RGN delivery were made using software available in the eDystrophin online database (http://edystrophin.genouest.org).

**Figure 5:**
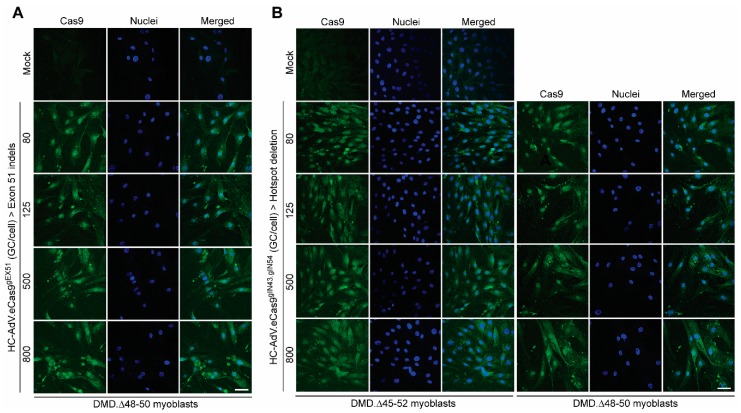
Detection of eCas9.4NLS expression in DMD myoblasts after “all-in-one” HC-AdV transduction of single and dual RGNs. (**A**) Immunofluorescence microscopy analysis on DMD.Δ48–50 myoblasts transduced with CD46-targeting HC-AdV particles encoding RGN-targeting *DMD* exon 51. (**B**) Immunofluorescence microscopy analysis on DMD.Δ45–52 and DMD.Δ48–50 myoblasts transduced with CD46-targeting HC-AdV particles co-expressing RGNs specific for sequences in *DMD* introns 43 and 54. The confocal microscopy analyses were carried out at 3 days post-transduction in the presence of the DNA dye DAPI (Nuclei staining). Mock-transduced cells served as negative controls. The vector genome copies per cell (GC/cell) applied are indicated. Magnification bar in both panels corresponds to 50 µm.

**Figure 6:**
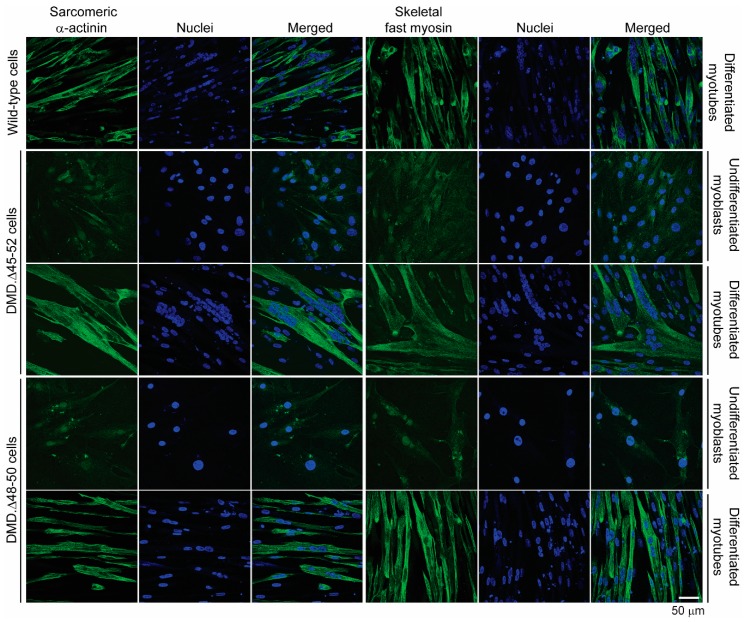
Assessment of the differentiation capacity of DMD myoblasts transduced with *DMD*-editing HC-AdV particles. DMD.Δ45–52 and DMD.Δ48–50 myoblasts were transduced with CD46-targeting HC-AdV.eCas9^gIN43.gIN54^ particles at an MOI of 500 genome copies per cell (GC cell^−1^). Sarcomeric α-actinin and skeletal fast myosin immunofluorescence microscopy analyses were performed before and after myoblasts differentiation at 3 days and 8 days post-transduction, respectively. Nuclei were labeled with the DNA dye DAPI. Mock-transduced wild-type human myotubes served as positive controls for the detection of the skeletal muscle differentiation markers sarcomeric α-actinin and skeletal fast myosin.

Encouraged by this cumulative data informing of a broader dosage window offered by the HC-AdV platform, we next generated CD46-targeting HC-AdVs encoding high-specificity RGNs consisting of optimized Cas9 and gRNA components. In particular, we assembled CD46-targeting HC-AdV.eCas9^gEX51^ and HC-AdV.eCas9^gIN43.gIN54^ particles. The purified vector preparation titers corresponding to the former and latter vector were, respectively, 1.0 × 10^12^ GC mL^−1^ and 0.6 × 10^12^ GC mL^−1^. These titers were in the same order of magnitude of that corresponding to the CD46-targeting HC-AdV.eCas9 vector lacking gRNA expression units whose titer was 1.7 × 10^12^ GC mL^−1^. The vector HC-AdV.eCas9^gEX51^ expresses the RGN complex eCas9.4NLS:opt-gRNA^EX51^ for testing *DMD* reading frame repair through targeted indel formation at exon 51 (Figure 4A). The vector HC-AdV.eCas9^gIN43.gIN54^ co-expresses the RGN complexes eCas9.4NLS:opt-gRNA^IN43^ and eCas9.4NLS:opt-gRNA^IN54^ for investigating *DMD* reading frame repair via targeted deletion of the >500 kb major mutational hotspot region (Figure 4B). Structural analysis of recombinant vector genomes isolated from control (i.e., HC-AdV.eCas9) and *DMD*-editing HC-AdV particles (i.e., HC-AdV.eCas9^gEX51^ and HC-AdV.eCas9^gIN43.gIN54^) ascertained their genetic integrity (Supplementary Figure S3). Moreover, transduction of human myoblasts with the tropism-modified HC-AdVs encoding *DMD*-targeting RGNs led to eCas9.4NLS synthesis in the vast majority of the target cells (Figure 5). These results are consistent with the high transduction frequencies measured in cultures of human myoblasts exposed to tropism-modified AdVs encoding the reporter EGFP (Figure 1C). Importantly, patient-derived DMD.Δ45–52 and DMD.Δ48–50 myoblasts exposed to tropism-modified HC-AdVs retained their differentiation capacity, as determined by immunofluorescence microscopy analyses of late skeletal muscle markers (Figure 6).

**Figure 7:**
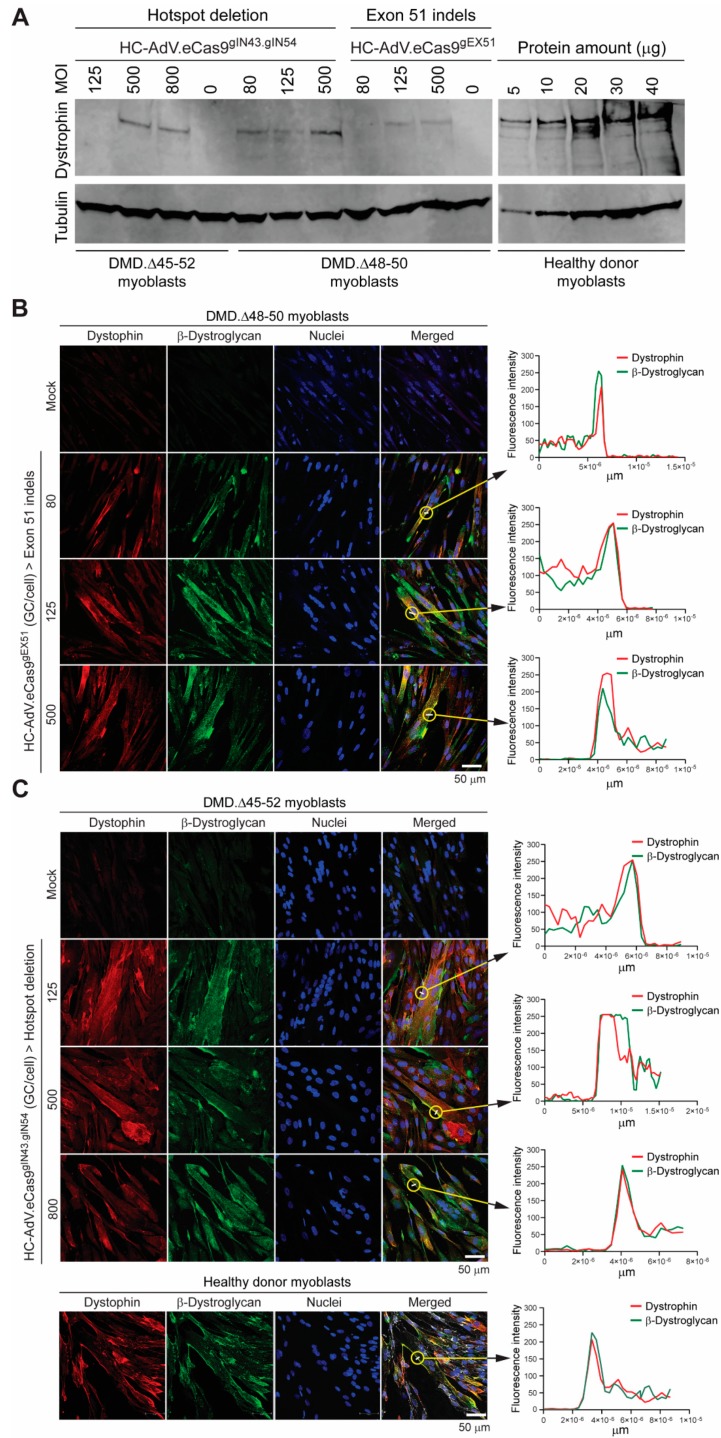
Assessing dystrophin rescue in DMD muscle cells after “all-in-one” HC-AdV transduction of single and dual RGNs. (**A**) Direct detection of dystrophin in populations of *DMD* edited muscle cells by western blot analysis. Western blotting was performed on myotubes differentiated from myoblasts with the Δ45–52 and Δ48–50 genotypes previously transduced with HC-AdV.eCas9^gIN43.gIN54^ or with HC-AdV.eCas9^gEX51^ at the indicated MOIs. To serve as reference, western blotting was also carried out on various protein amounts from healthy donor myotubes. An antibody recognizing α/β-tubulin provided for a protein loading control. (**B**) Immunofluorescence microscopy analyses on muscle cells edited by HC-AdV particles encoding RGNs targeting *DMD* exon 51. Dual-color confocal microscopy for dystrophin and β-dystroglycan was performed on myotubes differentiated from DMD.Δ48–50 myoblasts transduced with HC-AdV.eCas9^gEX51^ at the indicated MOIs. (**C**) Immunofluorescence microscopy analyses on muscle cells edited by HC-AdV particles encoding dual RGNs targeting *DMD* introns 43 and 54. Confocal microscopy for dystrophin and β-dystroglycan was done on myotubes differentiated from DMD.Δ45–52 myoblasts transduced with HC-AdV.eCas9^gIN43.gIN54^ at the indicated MOIs. Healthy donor (wild-type) myotubes served as positive controls; GC/cell: genome copies per cell. In panels **B** and **C**, co-localization of dystrophin and β-dystroglycan molecules across the plasma membrane of gene-edited DMD myotubes was confirmed by measuring the fluorescence intensities of dystrophin- and β-dystroglycan-specific signals at the encircled locations. Nuclei were labeled with the DNA dye DAPI.

Next, we asked whether HC-AdV.eCas9^gEX51^ and HC-AdV.eCas9^gIN43.gIN54^ could, after transduction and subsequent differentiation of DMD patient-derived myoblasts, directly lead to the detection of Becker-like dystrophins in the resulting bulk muscle cell populations, i.e., without having to resort to additional experimental maneuvers, e.g., selecting *DMD* edited clones or enriching for sub-populations exposed to high RGN concentrations. To this end, myoblasts with *DMD* genotypes Δ45–52 and Δ48–50 (i.e., DMD.Δ45–52 and DMD.Δ48–50 myoblasts, respectively), were transduced with the dual RGN-encoding vector HC-AdV.eCas9^gIN43.gIN54^. Parallel cultures of DMD.Δ48–50 myoblasts were also transduced with the single RGN-encoding vector HC-AdV.eCas9^gEX51^. Western blot analysis revealed the synthesis of Becker-like dystrophins exclusively in cultures of differentiated muscle cells whose progenitors had been subjected to each of the tropism-modified HC-AdVs (Figure 7A). Importantly, the differential electrophoretic migration observed between the de novo generated dystrophin species and between these species and the wild-type protein (Figure 7A) was consistent with their expected relative molecular weights (Figure 4A,B).

As previously mentioned, in striated muscle cells, dystrophin anchors the cytoskeleton to the DGC, a large sarcolemma-embedded macromolecular complex that, amongst other members, contains the transmembrane protein β-dystroglycan. Through its linkage to a cysteine-rich region near the C-terminus of dystrophin, β-dystroglycan is stabilized and properly assembled within an intact DGC. However, in the absence of functional dystrophin molecules, similarly to other DGC components, β-dystroglycan is dislodged from the plasma membrane and has a shorter half-life [57]. Thus, to further assess the rescue of dystrophin protein synthesis, we sought to determine whether HC-AdV-induced *DMD* gene editing could result in the stabilization and proper relocation of β-dystroglycan to the plasma membrane of differentiated muscle cells. Consistent with the western blot analysis, confocal immunofluorescence microscopy readily detected dystrophin in myotubes differentiated from DMD.Δ48-50 myoblasts transduced with HC-AdV.eCas9^gEX51^ (Figure 7B) as well as in myotubes differentiated from DMD.Δ45-52 and DMD.Δ48-50 myoblasts transduced with HC-AdV.eCas9^gIN43.gIN54^ (Figure 7C and Supplementary Figure S4, respectively). Moreover, independently of *DMD* cell genotypes and gene editing strategy, DMD myotubes expressing Becker-like dystrophins also had a substantially higher accumulation of β-dystroglycan when compared to that detected in myotubes differentiated from mock-transduced DMD myoblasts (Figure 7B,C and Supplementary Figure S4). Significantly, similarly to myotubes from a healthy donor, in DMD myotubes containing gene-edited nuclei, the highest co-localization of dystrophin and β-dystroglycan was generally found along the plasma membrane (Figure 7B,C, graph insets).

**Figure 8:**
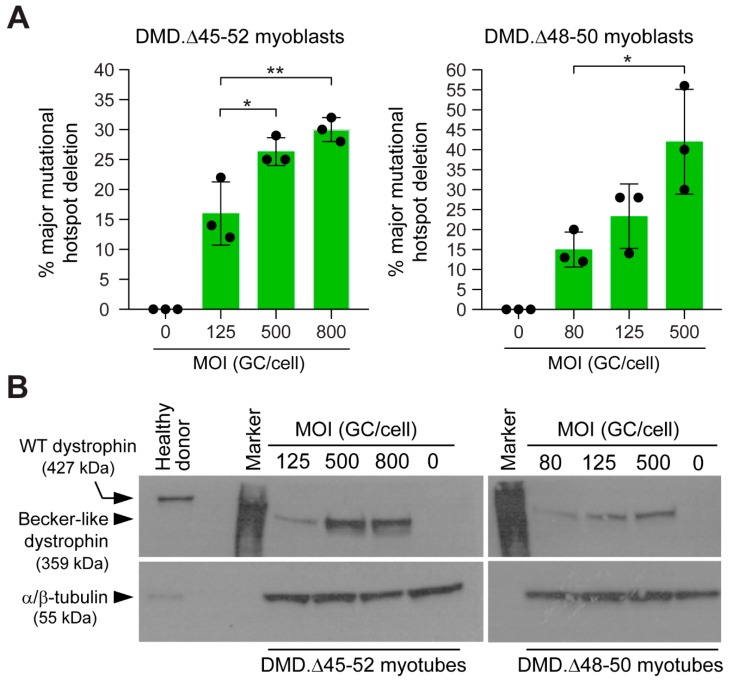
“All-in-one” HC-AdV transduction of RGN multiplexes for *DMD* repair through long-range genomic deletions. (**A**) Determining major *DMD* hotspot deletion frequencies. qPCR analysis on genomic DNA from DMD.Δ45–52 and DMD.Δ48–50 myoblasts transduced with HC-AdV.eCas9^gIN43.gIN54^ at the indicated multiplicities of infection (MOI). GC/cell: vector genome copies per cell. At 3 days post-transduction, mock-transduced and vector-transduced cells were sub-cultured, and total cellular DNA was isolated at 7 days post-transduction for qPCR analyses. Parallel cultures of mock-transduced cells provided for negative controls. Bars and error bars correspond to mean ± SD from three independent biological replicates. Each data point is the average of three technical replicates. Significance among datasets was calculated by one-way ANOVA followed by Tukey’s test for multiple comparisons. *p < 0.05; **p < 0.01; *p* ≥ 0.05 was considered non-significant. (**B**) Detection of dystrophin in populations of *DMD* edited muscle cells by western blot analysis. Western blotting was carried out on myotubes differentiated from *DMD*-defective myoblasts transduced with HC-AdV.eCas9^gIN43.gIN54^ at the indicated MOIs.

The assembly of in-frame *DMD* transcripts through the deletion of the major mutational hotspot region conveys several advantages when compared to exon-targeted approaches. Firstly, it can cover a wider range of DMD patient genotypes. Secondly, by targeting large introns, it provides flexibility for identifying suitable gRNAs while avoiding introducing out-of-frame indels or de novo epitopes within coding DNA. Thus, in follow-up gene-editing experiments, we focused on determining targeted deletion frequencies of the major *DMD* mutational hotspot following HC-AdV.eCas9^gIN43.gIN54^ transductions. To this end, DMD.Δ45–52 and DMD.Δ48–50 myoblasts were exposed to various MOIs of dual RGN-encoding HC-AdV.eCas9^gIN43.gIN54^ particles. Next, a qPCR assay designed for quantifying the chromosomal junctions formed after co-expression of RGNs eCas9.4NLS:opt-gRNA^IN43^ and eCas9.4NLS:opt-gRNA^IN54^ was performed at 7 days post-transduction. The cumulative data from these experiments revealed a dose-dependent increase in the frequencies of targeted DNA deletions (Figure 8A). The highest frequencies of *DMD* alleles lacking the major mutational hotspot region (42% ± 13%) was measured in DMD.Δ48-50 myoblasts transduced with HC-AdV.eCas9^gIN43.gIN54^ at an MOI of 500 GC cell^−1^ (Figure 8A). Importantly, after sub-culturing, myoblasts that had been transduced with HC-AdV.eCas9^gIN43.gIN54^ went on to differentiate into syncytial myotubes in which the build-up of Becker-like dystrophin molecules, as assessed by western blot analysis (Figure 8B), largely mirrored the vector dose–dependent *DMD* repair frequencies measured by qPCR (Figure 8A).

In conclusion, we report that the HC-AdV platform offers broader vector dose ranges than those consented by its previous, second-generation, AdV counterpart. Moreover, further strengthening the versatility of this gene delivery platform, we demonstrate that tropism-modified HC-AdVs induce robust *DMD* gene repair in human myogenic cells after “all-in-one” delivery of single or multiple programmable nucleases based on optimized high-specificity RGNs. Indeed, correlating with the high frequencies of targeted genomic deletions removing *DMD* mutations in patient-derived myoblasts, Becker-like dystrophins were directly detected in bulk muscle cell populations. Hence, selecting *DMD-*edited cells or RGN-exposed cell fractions prior to dystrophin expression assessments was dispensable regardless of whether the *DMD* gene editing approach was based on single or dual RGN delivery. Importantly, the amounts of Becker-like dystrophins produced in unselected muscle cell populations sufficed for stabilizing and relocating β-dystroglycan to its proper location at the plasmalemma, which, in turn, suggests DGC assembly in syncytia containing edited myonuclei. Taken together, these data strengthen the rationale for developing genetically retargetable HC-AdVs aiming at permanent and safe rescue of endogenous *DMD* expression after transient delivery of optimized gene-editing reagents. Towards that end, the HC-AdV platform should further permit screening and identifying gene-editing reagents and procedures that are minimally disruptive to the genome of human cells.

## 4. Discussion

Research directed at tacking DMD is moving at a sustained pace and includes transplantation of cells with myoregenerative capacity and viral vector transduction of RGNs into dystrophic animal models [18,58,59]. So far, the vast majority of the latter studies involved co-administrations of AAVs encoding different Cas9 nucleases and respective gRNAs into murine and canine models [58]. Notwithstanding their fairly short-term monitoring (typically <2 months), the cumulative data from these reports indicate that AAV RGN delivery is generally well tolerated and, importantly, can improve striated muscle function in dystrophic mice [24,25,26]. A recent 1-year follow-up study has, however, uncovered immune responses against the small *Staphylococcus aureus* Cas9 nuclease and capsids of AAV serotypes (i.e., AAV-8 and AAV-9) in adult but not newborn dystrophic mice [32]. In addition to this conceivably surmountable bottleneck, the same study also disclosed a potentially more insidious outcome in the form of prevalent integration of Cas9-encoding AAV DNA at target sequences in transduced muscle tissues [32]. A similar phenomenon was thoroughly analyzed in another recent report in which portions of AAV inverted terminal repeats linked to full-length or truncated transgenes were invariably found at RGN target sites in various mouse tissues, including at *Dmd* exons 51 and 53 in dystrophic skeletal muscle [33]. Besides raising concerns associated with the permanency of RGN-coding sequences in transduced cells, these results extend earlier data documenting that recombinant AAV genomes are prone to homology-independent chromosomal insertion at random and targeted DSBs in vitro [27,28,29] and at zinc-finger nuclease-induced DSBs in murine livers [30,31]. Hence, on the one hand, these findings stress the importance of closely monitoring the accuracy of AAV gene-editing procedures and, on the other, forcefully expand the range of *DMD*-targeting agents, especially those that, like AAV, do not carry viral genes.

Previous research from our laboratory has demonstrated that, in contrast to free-ended viral and non-viral vector DNA, protein-capped AdV genomes are refractory to chromosomal DNA integration in human cells, even in the presence of targeted DSBs [51]. Moreover, subsequent experiments revealed that retargeted second-generation AdVs encoding conventional dual RGNs addressed to target sites in *DMD* introns 43 and 54 corrected up to 18% of *DMD* alleles in patient-derived myoblasts [22]. In the current work, after demonstrating that HC-AdVs are significantly less cytotoxic than second-generation AdVs, we incorporated into retargeted HC-AdV particles one or two gRNA expression units, together with a transgene designed for maximizing expression and nuclear enrichment of the high-specificity eSpCas9(1.1) nuclease [53]. These HC-AdV.eCas9^gEX51^ and HC-AdV.eCas9^gIN43.gIN54^ particles were assembled for removing, through NHEJ-based gene editing, *DMD* mutations present in 13% and >60% of the patient population, respectively [6]. Regardless of the NHEJ-based gene editing strategy investigated, RGN delivery through HC-AdV transduction led to the accumulation of Becker-like dystrophins and β-dystroglycan at the plasmalemma of unselected muscle cell populations. Notably, HC-AdV.eCas9^gIN43.gIN54^ expressing optimized high-specificity RGNs targeting the aforementioned *DMD* introns 43 and 54 sequences corrected up to 42% ± 13% of *DMD* alleles in patient-derived myoblasts.

Clearly, either postulated or experimentally validated in model systems, ex vivo and in vivo DMD genetic therapies have their own sets of pros and cons [18]. Yet, in view of the challenging and multifaceted nature of DMD (e.g., size of affected gene and tissues and stage-specific phenotypes), these approaches might become, to some extent, complementary. Hence, diversifying DMD-directed research lines and attendant tools holds particular significance.

HC-AdVs aggregate a unique set of features, i.e., vast packaging capacity (up to 36 kb), lack of viral genes, strict episomal nature and efficient transduction of dividing and terminally differentiated cells [19]. These features make these viral vectors suitable for investigating ex vivo and in vivo DMD genetic therapies based on fast-evolving gene-editing principles and technologies. Genetically retargetable HC-AdVs should be particularly useful for assessing *DMD* gene-editing tools and strategies in human CAR-negative stem/progenitor cells of muscle and non-muscle origins with myoregenerative capacity. Of note, the extent of RGN activity is in part determined by higher-order chromatin conformations [60,61]. Hence, the broad tropism of CD46-targeting HC-AdVs should permit (i) determining wanted and unwanted genome-modifying events resulting from the interaction of specific gene editing reagents with human (epi)genomes in different myogenic stem/progenitor cells and (ii) performing muscle cell biology studies under physiological and pathologic conditions. Related with the latter aspect, it is known that in-frame *DMD* exon deletions can confer phenotypes varying from asymptomatic to DMD-like [62,63]. Experimental evidence indicates that this phenotypic heterogeneity depends, at least in part, on the disruption of the regular phasing and completeness of the coiled-coil-forming spectrin-like repeats of dystrophin [63]. Thus, HC-AdVs should equally expedite structure-function dissection of specific dystrophin variants through the generation of “disease-in-a-dish” cellular models based on wild-type and *DMD*-edited myogenic cells with isogenic genetic backgrounds.

In conclusion, in the present work, we have established genetically retargetable HC-AdVs as a robust and flexible platform for *DMD* gene repair through the testing of “all-in-one” transfer of single or dual high-specificity RGNs into human muscle progenitor cells. Following from these data, HC-AdVs open the perspective for the effective assessment of the increasing number of gene-editing reagents and procedures in human cells and animal models.

## Supplementary Materials

The following are available online at https://www.mdpi.com/2073-4409/9/4/869/s1, Supplementary Table S1, oligonucleotides used for optimized gRNA assembly and corresponding target sites; Supplementary Table S2, primer pairs and composition for qPCR mixtures used for the quantification of genomic deletions; Supplementary Table S3, qPCR cycling parameters used for the quantification of genomic deletions; Supplementary Figure S1, quality control of qPCR amplicons used for quantifying long-range genomic deletions induced by dual RGN-encoding HC-AdV particles; Supplementary Figure S2, diagrammatic representation of wild-type and recombinant adenoviruses; Supplementary Figure S3, structural analysis of eCas9.4NLS-encoding HC-AdV genomes; Supplementary Figure S4, assessing dystrophin rescue in DMD muscle cells after “all-in-one” HC-AdV transduction of dual RGNs.
